# Climate variation explains a third of global crop yield variability

**DOI:** 10.1038/ncomms6989

**Published:** 2015-01-22

**Authors:** Deepak K. Ray, James S. Gerber, Graham K. MacDonald, Paul C. West

**Affiliations:** 1 Institute on the Environment (IonE), University of Minnesota, Saint Paul, Minnesota 55108, USA

## Abstract

Many studies have examined the role of mean climate change in agriculture, but an understanding of the influence of inter-annual climate variations on crop yields in different regions remains elusive. We use detailed crop statistics time series for ~13,500 political units to examine how recent climate variability led to variations in maize, rice, wheat and soybean crop yields worldwide. While some areas show no significant influence of climate variability, in substantial areas of the global breadbaskets, >60% of the yield variability can be explained by climate variability. Globally, climate variability accounts for roughly a third (~32–39%) of the observed yield variability. Our study uniquely illustrates spatial patterns in the relationship between climate variability and crop yield variability, highlighting where variations in temperature, precipitation or their interaction explain yield variability. We discuss key drivers for the observed variations to target further research and policy interventions geared towards buffering future crop production from climate variability.

## Abstract (web summary)

Agricultural crops are closely linked to the climate in which they grow, but the extent to which variability in weather influences yield is not well characterized. Here, Ray *et al*. find that climatic variation explains around a third of the variation in global crop yields, with important regional variations.

## Graphical abstract

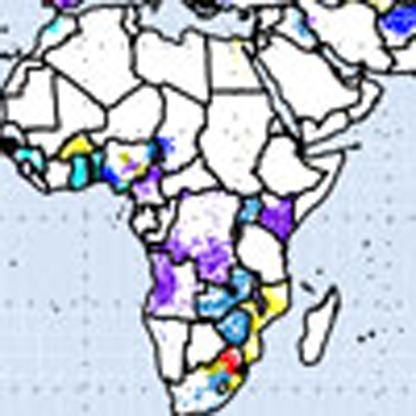

How mean historical and future climate change affects crop yields has received a great deal of attention^1^,^2^,^3^,^4^,^5^. However, how variations in climate impact crop yield, and how they vary over time, has received less attention^6^,^7^. This is important both to help us understand how climate and crop yields are linked over time and also for ensuring future food security. In particular, low-yield variability leads to stable farmer incomes^8^,^9^,^10^ and food supply^1^,^11^, and prevents price spikes that have disproportionate adverse impacts on the globally food-insecure who are mostly farmers^12^,^13^. In this study, we ask how much of the year-to-year variability in observed crop yields is associated with variations in climate across global croplands? Further, we investigate which climatic variables—those related to warmth and growing season length, or those related to rainfall and moisture availability—best explain variations in yield across the world?

Previous analyses that have examined how crop yields and climate were related^3^,^14^,^15^,^16^,^17^ have typically used national and regional data. For example, global studies are typically at the country scale^1^, and provide little insight on the spatial patterns of the within-country impacts. In contrast, analysis at the subnational^16^, sub-subnational^2^ or local sites is available for specific countries only, and thus, provide little insight on global patterns. Our study uses newly available temporal geospatial data on crop harvested area and yields of four major crops (maize, rice, wheat and soybean) across 13,500 different political units of the world^12^,^13^—a major (>50x) increase in the level of spatial detail from previous analyses that examined how crop yields and climate were related^3^,^14^,^15^,^16^,^17^. Similar to other studies^1^,^2^, we examine the recent historical period (1979–2008) but across these 13,500 different political units of the world^12^,^13^. The increased spatial resolution helps to identify where, and how strongly, climate variability is correlated with variations in crop yield in each of these political units. Given multiple breadbaskets across the globe and globally traded commodities, our study provides a consistent investigation of the differences both within and across regions.

To examine how observed variations in yields were related to climate variations, we used the Climate Research Unit’s (CRU)^18^ gridded monthly data, and then re-mapped the data to the ~13,500 political units where yield was measured. We explored a range of statistical models relating observed de-trended variations in temperature and precipitation during a crop’s growing season and annual conditions to the observed de-trended variations in yields at each political unit. Next we selected the ‘best-fit’ model, and then conducted *F*-tests to determine the goodness-of-fit of the selected model against the null model that assumes random climate variability. We conducted this analysis at each of the tracked 13,500 political units to draw conclusions on how much of the crop yield variability was explained by climate variability.

Different aspects of climate variability—temperature, precipitation, and the interaction of the two—may affect crop growth and resultant productivity disproportionately. We classified how yield variability was related to either normal or extreme fluctuations in temperature or precipitation variability—or their interactions. Here, linear and squared terms represent normal and extreme variation, respectively, for example^1^,^5^,^16^. The ‘best-fit’ model at each political unit was classified into one of seven broad categories and then mapped globally: models where the yield variability was explained by (i) normal temperature or (ii) normal precipitation variations, but not both; models where the yield variability was explained by (iii) normal and extreme temperature or (iv) normal and extreme precipitation variations, but not both; (v) where yield variability was explained by extreme temperature or (vi) extreme precipitation variations, but not both; and (vii) temperature and precipitation terms and their combinations due to interactions between temperature and precipitation. We further developed reduced models of temperature and precipitation and mapped them at each political unit. The resulting global maps, which identify where and to what degree normal and extreme climate variability explains yield variability, and quantifies them, can be used to target research into causal relations between yield and climate variability, and eventually policy interventions to stabilize farmer incomes and food supply.

Averaged globally over areas with significant relationships, we find that 32–39% of the maize, rice, wheat and soybean year-to-year yield variability was explained by climate variability. This translates into climate explained annual production fluctuations of ~22 million tons, ~3 million tons, ~9 million tons and ~2 million tons for maize, rice, wheat and soybean, respectively. Our spatially detailed assessment of the relationship between climate variability and yield variability shows distinct spatial patterns in the relative effects of temperature, precipitation and their interaction within and across regions.

## Results

### Yield variability

We first establish where and by how much crop yields varied within countries and then identify how much of the year-to-year variation in crop yields was explained by year-to-year variations in climate.

**Figure 1:**
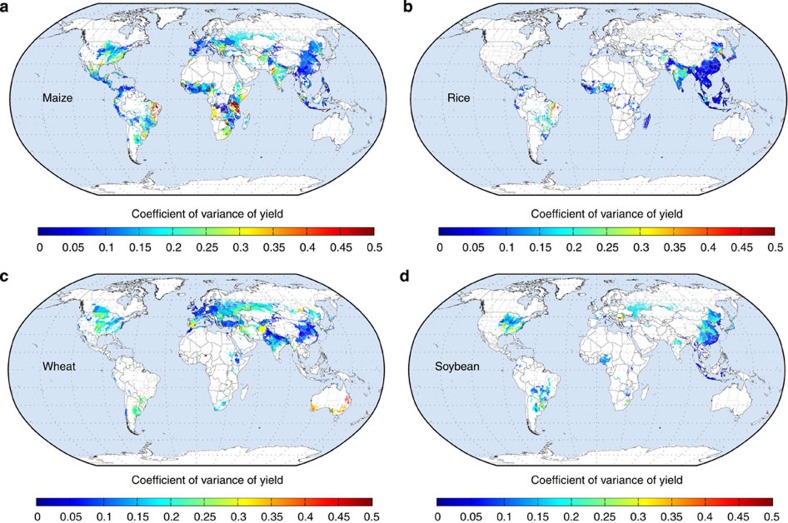
Coefficient of variation of crop yields over the entire study period. The ratio of the s.d. of yield over the 30-year period to the average yield over the same period. (**a**) maize, (**b**) rice, (**c**) wheat, (**d**) soybean (sample size of ~13,500 political units × 30 years per crop). White areas indicate where the crop is not harvested or analysed. Details on crop yields are given in reference 13.

In general the coefficient of variation, or yield variability normalized by mean yields (Fig. 1), has been lower in the top crop production regions of the world on account of their higher yields (Supplementary Fig. 1) and conversely higher in the areas of lower yields and of less consequence to global crop production but exceptions such as the Australian wheat belt exist. Over the last three decades, maize yields had a global average variability of ~0.9 tons/ha/year (s.d.), which corresponds to ~22% of the global average yields of ~4 tons/ha/year (Supplementary Fig. 1). The highest coefficient of variation—which indicates greatest relative variability—in maize yields was in areas outside the core maize grain belts, including northeastern Brazil and in parts of Africa, India, northeast Mexico and the southeast United States (Fig. 1). The global average rice yield variability (standard deviation) was ~0.5 tons/ha/year (or ~13% of average rice yields). The coefficient of variation in rice yields was similarly higher in more marginal rice-producing regions such as northeastern Brazil and central India. In contrast, some wheat regions with high coefficient of variation in yields such as in Australia and the Great Plains states of the United States (U.S.) are key global wheat breadbaskets (Fig. 1). The global average wheat yield variability (s.d.) was 0.4 tons/ha/year (~17% of average yields over the study period). In the top soybean production areas of the world such as in the Midwestern U.S. and Latin American countries, the coefficient of variation was low.

### Climate explained yield variability

**Figure 2:**
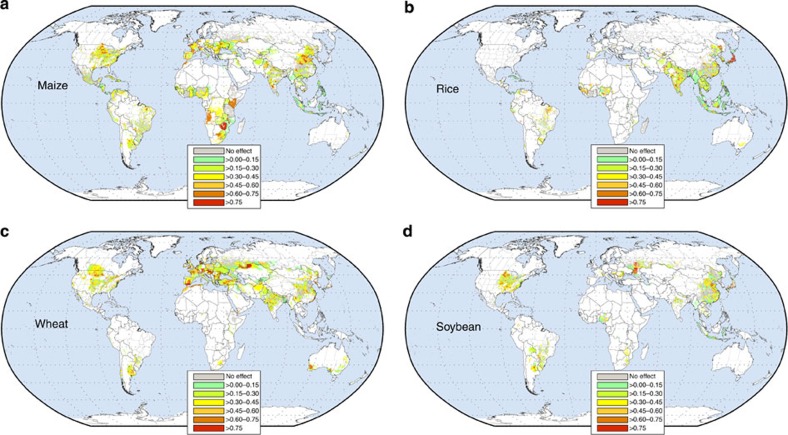
Total crop yield variability explained due to climate variability over the last three decades. A value of 1.0 implies that the entire variability in observed yields was explained by climate variability (coefficient of determination metric; sample size of ~13,500 political units × 30 years per crop). Similarly a value of 0.30–0.45 implies 30–45% of the variability in yields was explained by climate variability. We cutoff the range at 0.75 (or 75%) and above to a single categorical colour. No effect implies that at the *P*=0.10 level, there was no statistical difference between the best fit model and the null model in the political unit. White areas indicate where the crop is not harvested or analysed. (**a**) maize, (**b**) rice, (**c**) wheat, (**d**) soybean.

Not all crop growing regions showed statistically significant influence of year-to-year variations in climate on crop yield variability as determined from conducting *F*-tests (using a threshold of *P*=0.1; ~13,500 political units × 30 years sample size, Fig. 2). However, the vast majority of crop harvesting regions did experience the influence of climate variability on crop yields: ~70% of maize harvesting regions, ~53% of rice harvesting regions, ~79% of wheat harvesting regions and ~67% of soybean harvesting regions. The percentage of global total average production harvested over these regions and thus influenced by climate variability was ~78% of maize, ~52% of rice, ~75% of wheat and ~67% of soybean. In specific locations, within the top global crop production regions, climate variability accounted for >60% of the variability in a crop’s yield, though there were also political units where climate impacts have been statistically insignificant (Fig. 2). Where and how much of a crop’s yield varied on account of climate has been highly location- and crop–specific, and we describe this in greater detail in the subsequent sections.

Averaged globally over areas with significant relationships, we find that 32–39% of the maize, rice, wheat and soybean year-to-year yield variability was explained by climate variability (Supplementary Data). Climate variability in general explains rice yield variability the least.

### Regional variations in maize

Approximately 75% of the global maize production is concentrated in ~57% of the harvested areas comprising the American Midwestern region, central Mexico, southern Brazil, the maize belts of Argentina and China, parts of Western Europe and South Africa, and some areas of India and Indonesia. In these major maize grain belts, ~41% of the total year-to-year yield variability (0.8 tons/ha/year) was explained by inter-annual climate variability. Approximately 50% of global maize production is concentrated in a proportionally even smaller ~31% of high yielding maize belt comprising primarily two regions—the American Midwest and the Chinese Corn Belt—and in these two regions ~42% of the corresponding yield variability (0.9 tons/ha/year) was explained by climate variability.

In some specific political units within these maize breadbaskets, more than 60% of the yield variability has historically been related to climate variability including numerous counties of the U.S. Midwestern states, and in Shanxi, Hebei and Shandong provinces of China (Fig. 2a); political units with >75% of the yield variability explained by climate are also present, for example, many counties of Midwestern U.S.

When averaged over all the statistically significant maize harvested areas with climate variability impacts globally 39% of the yield variability was explained and in the top ten global maize producing nations we find the following (Supplementary Data): in the United States, France and Italy 41–49% of the observed maize yield variability can be explained by climate variability, whereas in South Africa it was ~50%, and in Argentina and China it was 32 and 44%, respectively (Fig. 2a and see Supplementary Data).

**Figure 3:**
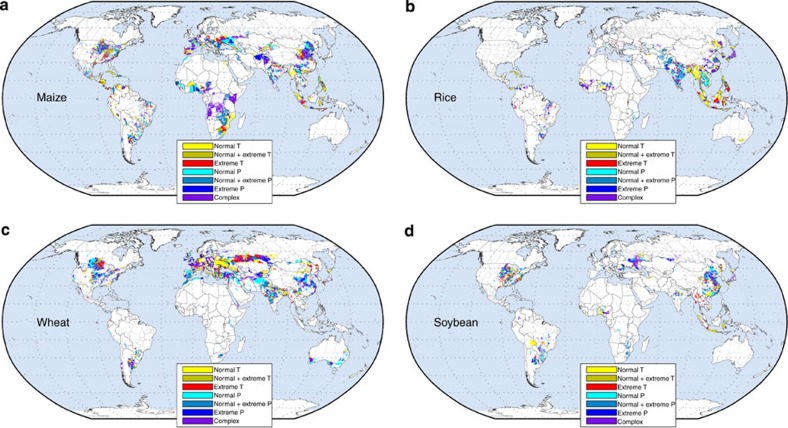
Selected models explaining crop yield variability classified into seven categories of temperature and precipitation variations. White areas indicate where the crop is not harvested or analysed. (**a**) maize, (**b**) rice, (**c**) wheat, (**d**) soybean. Regions where models with only normal temperature (T) terms are selected are shown in yellow colour; regions where models with normal and extreme temperature (T^2^) terms are selected are shown in tan colour; regions where models with only extreme temperature terms are selected are shown in red colours. Similarly, regions where models with normal, normal and extreme, and only extreme precipitation (P) terms are selected are shown in the maps with different shades of blue. Regions where models with both temperature, precipitation and their interactions terms were selected are shown in purple colour.

In the upper and eastern Midwest of the United States and Canada extreme temperature variability was more important, whereas in the central and western parts of Midwestern U.S. extreme precipitation variability explained maize yield variability in more counties (Fig. 3a); overall temperature variability was more important for explaining maize yields in the upper and eastern Midwest of the United States and precipitation variability was more important in the central and western Midwest (Supplementary Figs 2 and 3). Temperature variability influenced maize yield variability more in some colder countries such as in Canada, but also in some warmer countries such as Spain and Italy with within-country variations.

### Regional variations in rice

Approximately 75% of global rice production was from China, India and Indonesia. Averaged over all rice-harvesting areas with statistically significant climate influence (around 52% of global rice harvested areas), we estimate that yields have varied by ~0.1 tons/ha/year. Year-to-year climate variability explains ~32% of rice yield variability globally (Fig. 2b) with precipitation variability explaining more of the variability in South Asia and temperature variability more of the variability in Southeast and East Asia (Fig. 3b and Supplementary Figs 2 and 3). In some key rice producing nations, however, climate variability was more important: in 80% of the rice harvested areas in Japan climate variability was statistically significant. Averaged over these areas we find that ~79% of the rice yield variability (~0.2 tons/ha/year) was explained, whereas, in South Korea, ~47% of the yield variability was explained by climate variability (Fig. 2b). In both countries temperature variability was more important (Supplementary Figs 2 and 3). In India, China, Indonesia, Thailand, Brazil, Cambodia, Peru and Spain, 25–38% of the yield variability was explained by climate variability (Supplementary Data). As with maize, there are specific regions where >60% of yield variability was explained by climate variability such as in the central Indian states of Madhya Pradesh, Chhattisgarh and Karnataka (Fig. 2b).

### Regional variations in wheat

Approximately 75% of global wheat production came from ~66% of the harvested lands in the United States, Canada, Argentina, Europe, North Africa, India, China and Australia. In these highly productive wheat belts, ~36% of the year-to-year yield variability was explained by climate variability (Fig. 2c). Approximately 34–45% of the wheat yield variability in the United States, Canada, United Kingdom, Turkey, Australia and Argentina was explained by climate variability. To give an indication of the magnitude of this effect, the climate driven variability in the United States wheat yields equates to, on average, more than half the entire annual production of wheat in Mexico.

In the more productive regions of some countries the variability explained by climate was even higher. In the most productive Australian wheat belt (among the top 50% of global wheat producers) climate variability explained ~43% of the total yield variability and in parts of Western Australia it was > 60%. In Western Europe in the United Kingdom, France, Germany, Spain and Italy, climate variability explained ~31–51% of the wheat yield variability. In Eastern Europe and the former Soviet Republics such as the Russian Federation, Ukraine, Kazakhstan and in Hungary, 23–66% of the wheat yield variability was explained by climate variability and normal and extremes of temperature variability was important (Fig. 3c). Although temperature variability in Western Europe was in general more important, precipitation variability also explained part of the wheat yield variability with the exception of Spain where precipitation variability was the dominant factor (Fig. 3c and Supplementary Figs 2 and 3). In India and China, the top two global wheat producers, we detected statistically significant relationships in 71 and 62% of their wheat harvested lands and on average 32 and 31% of yield variability, respectively, was explained by climate variability. In China precipitation variability explained most of the variability; in India temperature and precipitation variability were equally important (Fig. 3c). Averaged globally, climate variability explained ~35% of the wheat yield variability.

### Regional variations in Soybean

Approximately 50% of the world’s total soybean production was harvested from ~42% of the land concentrated in only three countries: the United States, Brazil and Argentina. Adding soybean lands in India and China made up 75% of the top soybean production global areas. In general, the variability in soybean yield related to climate variability was higher in Argentina (~43% of yield variability of 0.5 tons/ha/year averaged over all areas with statistically significant climate influence, but ~47% when averaged over the most productive areas), followed by ~36% of the yield variability explained over all the statistically significant U.S. soybean areas. Approximately 26–34% of the yield variability in Brazilian, Indian and Chinese soybean yields was explained by climate variability with locations of substantially higher variability explained present in all countries (Fig. 2d).

## Discussion

We show how much of the year-to-year variability in crop yields was associated with climate variability within and across regions. As the demand for crops increases globally^19^ and productivity gains fail to keep pace with projected demands^12^, ensuring the stability of national food supplies and farmer livelihoods to variable production will be even more important. Low global food stocks in conjunction with fluctuation in agricultural production can, in particular, contribute to food price spikes^20^,^21^. Regions with high crop yield variability would disproportionately contribute to this effect especially if they are also the major breadbaskets of the world^20^,^21^. Even in regions with comparatively lower yields, fluctuations in crop production may impact the local food security. Our study is unique in giving a global spatially detailed account of where and by how much crop yields have varied and how much of this was driven by climate variability.

We found that there were numerous regions where climate variability explained more than 60% of the yield variability in maize, rice, wheat and soybean (Fig. 2). Many of these regions were in the most productive global areas such as Midwestern U.S. and the Chinese Corn Belt for maize, and Western Europe and Australia for wheat.

Our study identifies unique spatial patterns in the effects of temperature and/or precipitation variability on yields—for example, rice and wheat in India (Fig. 3b,c) as well as maize and soybean in the United States (Fig. 3a,d). Our simple classification of the prevailing relationships between climate and crop yields enables digging deeper into trends for particular regions. While relatively high resolution compared with past research our results are constrained by the resolution of the data, which is at the political unit and monthly climate data. Within political units, at specific field/subnational locations, the climate variability impact could be higher or lower.

The 32–39% of the yield variability explained by climate variability translates into large fluctuations in global crop production. For example, ~39% of the maize yield variability of 0.6 tons/ha/year explained by climate variability over 94 million ha translates into an annual fluctuation of ~22 million tons in global maize production over the study period. Similar climate variability driven average annual rice, wheat and soybean production variability is ~3, 9 and 2 million tons, respectively. These average fluctuations are similar to the total maize production of many Latin American and African countries or the total rice production of some Asian countries or total wheat production of some Eastern European countries. In some cases the impact of climate variability is higher in poorer regions such as in northeastern Brazil for maize, and Central India for rice. However, even in the most productive global areas such as wheat in Western Europe and maize in the United States Midwest the influence of climate variability on yield variability is very high and in specific political units >75%. The following section discusses our regional and continental findings in the context of previous smaller-scale research, which we use to help validate/corroborate our results and explore possible drivers.

In the North China Plains (provinces of Hebei, Henan, Shandong, Beijing, Tianjin and Shanxi) though crops are irrigated^22^, water availability is a major problem^23^. Maize is a summer crop in this region and monsoonal rainfall supplements river and groundwater irrigation. High growing season temperature is common. Hence, both the temperature and precipitation variability controls maize yield variability in the North China plains. To the west of the North China Plains, in the more arid Loess Plateau region, adaptation strategies to the arid climate and the coincidence of rainfall during the later stages of crop growth^24^ lead to normal and extreme temperature variability being a better explanation of maize yield variability in some areas of Gansu and Ningxia and all of Shaanxi. Although it may appear counter-intuitive that temperature variability would dominate for rainfed maize, it is consistent with findings for rainfed maize areas in the United States^25^ where extreme temperature was found to be a better predictor of maize grain yield due to its control on soil water demand and transpiration rates. In contrast, wheat is a winter crop and is highly dependent on irrigation in the North China Plains. What our analysis shows is more dependence on precipitation variability for wheat yields, which may be due to the direct controlling influence on surface irrigation water availability. In northeastern China (provinces of Heilongjiang, Jilin, Liolin) maize and soybean are not widely irrigated so precipitation variability was important, but rice is irrigated so temperature variability became more important.

In Japan almost all the paddy rice crop is irrigated^22^,^26^ and hence temperature variability was more important compared with precipitation variability. South Korean harvested rice is similarly mostly irrigated and thus temperature variability was more important for explaining rice yield variability. In Indonesia the variability in rice yield explained by climate variability is often low (in the 0 to 15% range only) and the explanation is on account of temperature variability^27^ except in some parts such as Central Java where precipitation variability is also important^28^. This is because rice is widely irrigated in Indonesia^22^,^29^.

In South Asia, especially northwest India, temperature variability influences wheat yield variability widely, similar to other findings^30^ but further south in central and south India precipitation variability in general is more important as between half and three-fourths of wheat is rainfed winter wheat compared with only a few percent in the northwest. Rice yield variability is more influenced by precipitation variability in India indicating the rainfed paddy growing conditions. In the more irrigated parts^22^ as in northwest India precipitation and temperature variability or only temperature variability was important. In the extreme southwestern parts of India similarly precipitation and/or temperature variability was important as this region receives very high rainfall. Temperature variability was the important factor for rice yield variability in Bangladesh due to high availability of water and intense irrigation controlling the influence of precipitation variability. In some of the highly irrigated rice areas in India such as areas of West Bengal state, and the Mahanadi system in northern Orissa, climate variability was not even statistically significant (Figs 2b and 3b).

In Australia wheat yield variability is largely explained by precipitation variability as the wheat is grown under rainfed conditions^22^ and in agreement with previous findings^31^,^32^; controlling for precipitation variability, however, temperature variability was also an important factor in explaining wheat yield variability^33^ especially in parts of Western Australia, South Australia and Queensland.

We found that maize yield variability is explained best by normal and extreme precipitation variability related to ENSO in many countries of Africa similar to previous findings as in Zimbabwe^34^, which in turn is related to sea surface temperature^35^. In South Africa maize is grown primarily in the Highveld region with drier conditions in the west and wetter conditions in the east^36^. Our analysis reflects these conditions with precipitation variability being more important in the drier west and temperature variability more important moving towards the wetter eastern provinces of South Africa’s Highveld. Moreover, high maize yield variability in South Africa has been a concern^36^; indeed, we found that climate variability explained >60% of maize yield variability in the Highveld region especially in the drier western parts of the Highveld of South Africa.

Elsewhere, as in Kenya, we found that maize yield variability was explained only by a complex relationship between both precipitation and temperature variability consistent with previous studies^37^. In Cameroon in West Africa and in northeastern Nigeria precipitation variability alone does not explain maize yield variability agreeing with previous findings^38^,^39^ because, while rain is beneficial for stable maize production, it also triggers nitrogen leaching from nutrient poor soils, leading to a negative feedback. In many of the other West African countries rainfall variability explains maize yield variability but analyses show that this was not the case everywhere and neither does climate variability explain maize yield variability in all countries here as farmers adopt various management strategies to overcome the high rainfall variability^40^. However, other than Nigeria, our analysis in West Africa was only at the country level and within-country explanatory skill was lost on account of the scale of the available yield statistics. Overall, precipitation variability is more important in sub-Saharan Africa, pointing to the predominantly rainfed system of maize cultivation^41^.

In most of the Eastern Europe and many regions of Western European countries, the effect of temperature variability in explaining wheat yield variability was more important as also found in previous regional and global studies (refs 42, 43, Fig. 3c). This is because of the continental climate of Eastern Europe, which causes a greater amplitude of temperature variability^44^. Our study shows that normal, both normal and extreme, and extreme temperature variability was important in explaining wheat yield variability. In Southern Europe and in the Mediterranean regions in addition to heat stress the water limiting conditions that are common^44^,^45^,^46^ resulted in precipitation variability also being important for wheat yield variability. The influence of climate variability on wheat yield variability was not statistically significant everywhere. Neither was the explained variability in statistically significant areas high everywhere. This was because farmers are already adapted, or adapting, to climate change^47^, which has made them also more adapted to variability. In the United Kingdom either precipitation variability or both temperature and precipitation variability explained ~45% of wheat yield variability; the precipitation variability is in turn related with the North Atlantic Oscillation^48^.

Maize is partly irrigated in France, but irrigation does not fully mitigate dry conditions^49^; hence precipitation variability is important and also because irrigated maize areas have only recently increased in area and thus historically precipitation variability could not be compensated as effectively as more recently. The net result is that in many maize areas of France historically both temperature and precipitation variability are important^50^.

In the United States climate variability was important especially in the Midwestern U.S. for maize yields. While in the upper Midwest temperature variability was more important, in the central Midwest precipitation variability was more important. In Nebraska, a U.S. Great Plains state with a prevalence of irrigated maize in its western part, temperature variability was more important than in the eastern parts where precipitation variability was more important. Many of the counties of the Great Plains states with dryland maize meet their crop water demands partly from irrigation^51^ and we identify large number of counties where both precipitation and temperature variability was important. In other rainfed maize-producing countries normal and extreme temperature conditions explained maize yield variability due to increased soil water demand that raised transpiration rates and vapor pressure deficits^25^,^52^. Overall only temperature variability explained maize and soybean yield variability in more harvested regions (~37 and 38%, respectively) compared with precipitation only explained regions (~31 and 36%, respectively); climate variability explained part of the yield variability in ~91% of the U.S. maize harvested areas and 82% of soybean harvested areas. Less adaptation of farmers to increasingly warmer temperatures may explain why in larger areas temperature variability was important^53^.

Only ~46% of the maize harvested regions of Mexico have crop yield variability influenced by climate variability (~27% of the yield variability was explained). Precipitation variability was more important overall, but pockets of regions where temperature variability was more important exists such as in Sinaloa where irrigated maize is important, and in Guerrero. Temperature variability explained maize yield variability also in most Central American countries. Further south in Brazil, precipitation variability was more important overall; in specific regions temperature variability is overall more important such as Mato Grosso state due to its wetter climate, although in ~23% of Brazil’s maize harvested areas both temperature and precipitation variabilities were important in explaining part of the maize crop yield variability. In Argentina both temperature and precipitation variabilities were equally important overall, though in specific locations temperature variability was more important presumably due to irrigated maize.

Although this is the most spatially detailed global assessment of the links between historical climate variability and yield done to date, our study has some limitations. For example, our estimation of crop yield variability due to climate variability may underestimate the importance of climate variability impacts at specific locations within political units. Future studies should investigate this problem at an even finer resolution globally, but this is challenging given historical yield data availability.

In some countries both crop yield and weather data may have quality issues^13^,^18^. Our study is based on yield data at the county/district/municipal/department or larger political unit level, so we used crop harvested area weighted gridded weather data for the political units. However, weather data from individual stations could give a distinct climate-yield response signal due to its very localized scale. To test this latter issue, we carried out a separate analysis using daily station data from ~100 U.S. counties^54^ that contributed to ~25% of total U.S. maize production. We found statistically significant correlation (*r*=0.54; *P*=0.001) between analyses conducted by the two different data sets. A stronger relationship is likely not present with station data analysis due to the sheer size of some political units and lack of complete coverage, which is present in gridded data (Supplementary Fig. 4). As our yield was measured at the political unit, the use of the harvested area weighted gridded weather data^18^ for each political unit is appropriate, similar to previous upscaling usage^1^, and the likely reason that we typically found a stronger statistical relationship with yields over time (Supplementary Fig. 5). In contrast, the use of station data would be appropriate if crop yields were measured at same sites or locally, and the direct use of gridded data then less appropriate without downscaling.

While climate variability is a significant factor and responsible for 32–39% of global crop yield variability, it is certainly not the only controlling factor^55^. Our study only considered broad precipitation and temperature effects though in an unprecedented spatial detail; however, there are myriad other factors that could influence climate-yield relationships, as informed by more local scale research. Our study does not consider factors such as changing cloud cover (and solar radiation), wind speed, surface ozone exposure^56^, or decomposed the basic climate variables of temperature and precipitation further into the timing of heat stress^57^, the timing of dry and wet spells^58^, or soil moisture^59^. We have also not considered the amplification or dampening of climate variability impacts via other agronomic challenges such as pest and pathogen infestation^60^ and irrigation^61^. Climate change may also have influenced how frequently crops are harvested^62^,^63^, for example, now allowing double cropping in hitherto colder single cropped regions, but we were unable to include such precision as the only globally available crop calendar^64^ was static, even though we updated it using the most recent information available (see Supplementary Fig. 6). Other factors to consider in future studies are altitudinal effects^65^ and the quality of crop yields^48^. What we have investigated is the influence of the variability of temperature and precipitation on crop yield variability. The unexplained yield variability includes the numerous agronomic challenges and decisions that farmers make each year such as the availability and use of agronomic inputs^57^, pest and pathogen infestations^60^,^66^, soil management^66^,^67^, irrigation^61^, distribution of varied crop maturity types^68^, socio-economic conditions^55^,^63^,^69^ and political or social strife^13^.

Our study therefore is an initial assessment to identify locations worldwide where historically climate variability has been relatively important in explaining crop yield variability. From the perspective of stabilizing farmer incomes and national food supply and security, this new high-resolution information at the global scale should help direct further research and policy more effectively to those regions where climate variability poses the greatest risk and provide leverage points^70^ in the most critical regions. If climate variability is predicted to increase in the same regions where climate variability historically explained most of the crop yield variability, strategies to stabilize crop production should be prioritized to ensure stable future crop production and prevention of future food price spikes. The high-resolution models that we have built may be used to evaluate future climate-related yield variability research, provide cross-comparison against the results of crop simulation models and address alternate factors contributing to the spatial heterogeneity in climate-yield response.

## Methods

### Modelling set-up

Further details regarding the data used are given in Supplementary Methods 1. To determine how much of the variability in crop yields was explained by climate variability, we first detrended the crop yield and climate variables—temperature and precipitation—following^1^ (see the example in Supplementary Fig. 7) over the period 1979–2008. Note that we use two forms of temperature and precipitation—the seasonal or growing season average value and the average conditions 12 months before harvest or the annual value to account for antecedent conditions. This resulted in four different combinations of detrended climate variables, and as we used both the linear and squared forms of seasonal and annual temperature and precipitation there was a total of eight forms of climate variables. We used these detrended variables in different combinations to linearly regress with the detrended crop yields at each of the 13,500 political units. To avoid over-fitting we limited our analysis to a total of 27 combinations of climate variables, resulting in 27 regression equations, to capture the relationships between climate variability and crop yield variability at each political unit and of the basic form:




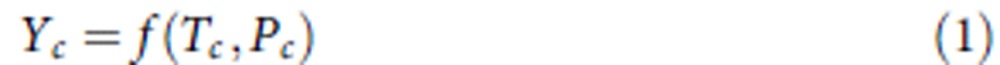




where *Y*_c_ is the observed set of detrended crop yields for crop ‘c’ in units of tons/ha/year at each political unit;

In equation 1, *T*_c_ can represent for crop ‘c’ at a given political unit the temperature associated with the main growing season^64^ or the temperature for 1 year before the crop’s harvest to capture antecedent conditions. *P*_c_ similarly is the precipitation for the main growing season for the crop ‘c’ for the political unit and for 1 year before the crop’s harvest. The function f is limited to linear and quadratic forms of these two detrended meteorological parameters, as is common practice in studies correlating climate and agricultural production^1^,^5^,^16^. The terms included in each of the 27 regression equations and their classification are provided in the Supplementary Table and further details are given in Supplementary Methods 2.

### Statistical tests

The generated regression equations at each political unit, for example, ~13,500 sets of 27 equations per crop, were statistically tested next. We first identified which functional form of *Y*_c_=*f*(*T*_c_, *P*_c_) from the set of 27 equations at each political unit fit the data best using the Akaike Information Criterion (AIC), which penalizes equations with more terms. However, because the model that best fits the data may be no better than a random climate (null model), we conducted *F* tests at the *P*=0.10 level to determine whether the chosen model was significantly better than the null model. In 21–47% of the global crop-harvested areas, we found that the chosen model was no better than the null model at the *P*=0.10 significance level. Thus, in the remainder 53–79% of global crop harvested areas yield variability is significantly influenced by climate variability over the study period and our reported numbers are averages over these areas.

Using the statistically significant model with the best functional representation, we next determined the coefficient of determination (*r*^2^) or explanatory power of the complete model, and the reduced models containing only temperature and only precipitation terms. The residual is the unexplained yield variations.

The 30-year study period average harvested area and yield information at each subnational location was used together with the observed coefficient of determination for computing national and global harvested areas weighted averages. Global and country-specific numbers are averaged only over those 53–79% of global crop harvested areas where the statistical models were significant.

### Model bias and sensitivity

As a simple assessment of model bias, we performed a bootstrapping exercise to assess the influence of including specific combinations of years (80% of the years selected at each iteration) in our data on the overall yield predictions (using a test set of 20% of the years) for each political unit, which we standardized as the ratio of the average bias from the 99 repetitions to the average of the crop yields for the study period in each political unit (Supplementary Fig. 8). This is analogous to a leave-group-out cross validation approach used to examine uncertainty in model selection. Locations of models with more restrictive *P* cutoff values (*F*-tests) at *P*=0.01 and *P*=0.05 are shown in Supplementary Fig. 9. In general, even though we used a less-restrictive *P* value of 0.1, the models selected generally were significant at *P*=0.05 or less.

## Author contributions

D.K.R. led the effort to design, conduct, analyze and write this report. J.S.G., G.K.M., P.C.W. contributed to statistical designing and writing. All authors analyzed and commented on the results.

## Additional information

**How to cite this article**: Ray, D. K. *et al*. Climate variation explains a third of global crop yield variability. *Nat. Commun.* 6:5989 doi: 10.1038/ncomms6989 (2015).

## Supplementary Material

Supplementary Information Supplementary Figures 1-9, Supplementary Table 1, Supplementary Methods and Supplementary References

Supplementary Dataset Coefficient of determination (R^2^) per crop-country combination averaged over significant areas
