# Supplementary material for: Climate variation explains a third of global crop yield variability
Source: Nat Commun. 2015 Jan 22;6:5989. doi: 10.1038/ncomms6989 (PMC4354156; doi:10.1038/ncomms6989)
Supplement: Supplementary Information — Supplementary Figures 1-9, Supplementary Table 1, Supplementary Methods and Supplementary References [file ncomms6989-s1.pdf]

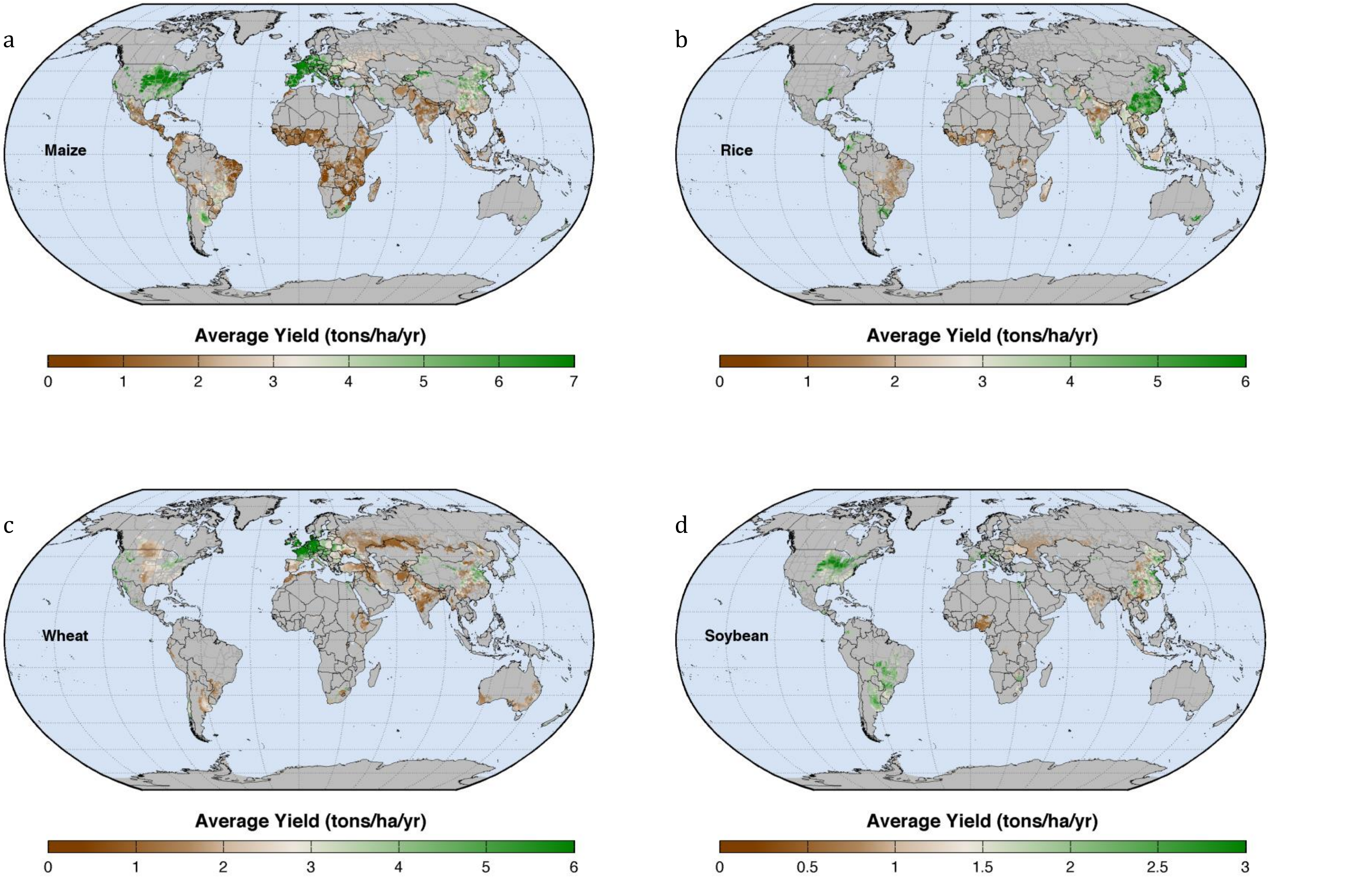

Supplementary Figure 1. **Average crop yields.** (a) Maize, (b) Rice, (c) Wheat, (d) Soybean. Averages are over the entire study period of 1979-2008. Note that the legend varies with the crop. Gray values indicate regions where crop is not harvested.

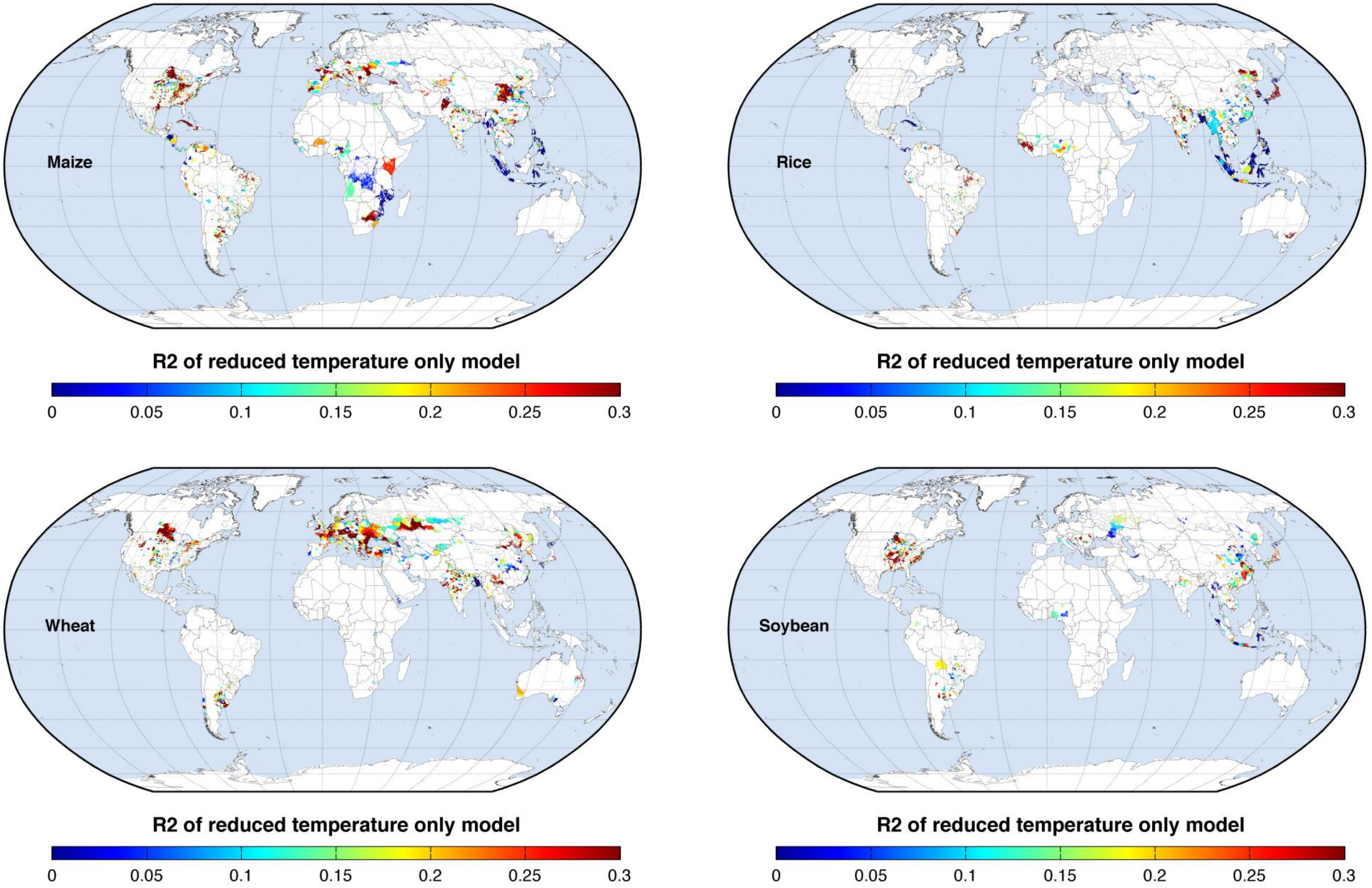

Supplementary Figure 2. **Coefficient of determination of reduced temperature models (r_T^2).** White areas indicate where crop is not harvested or analysed.

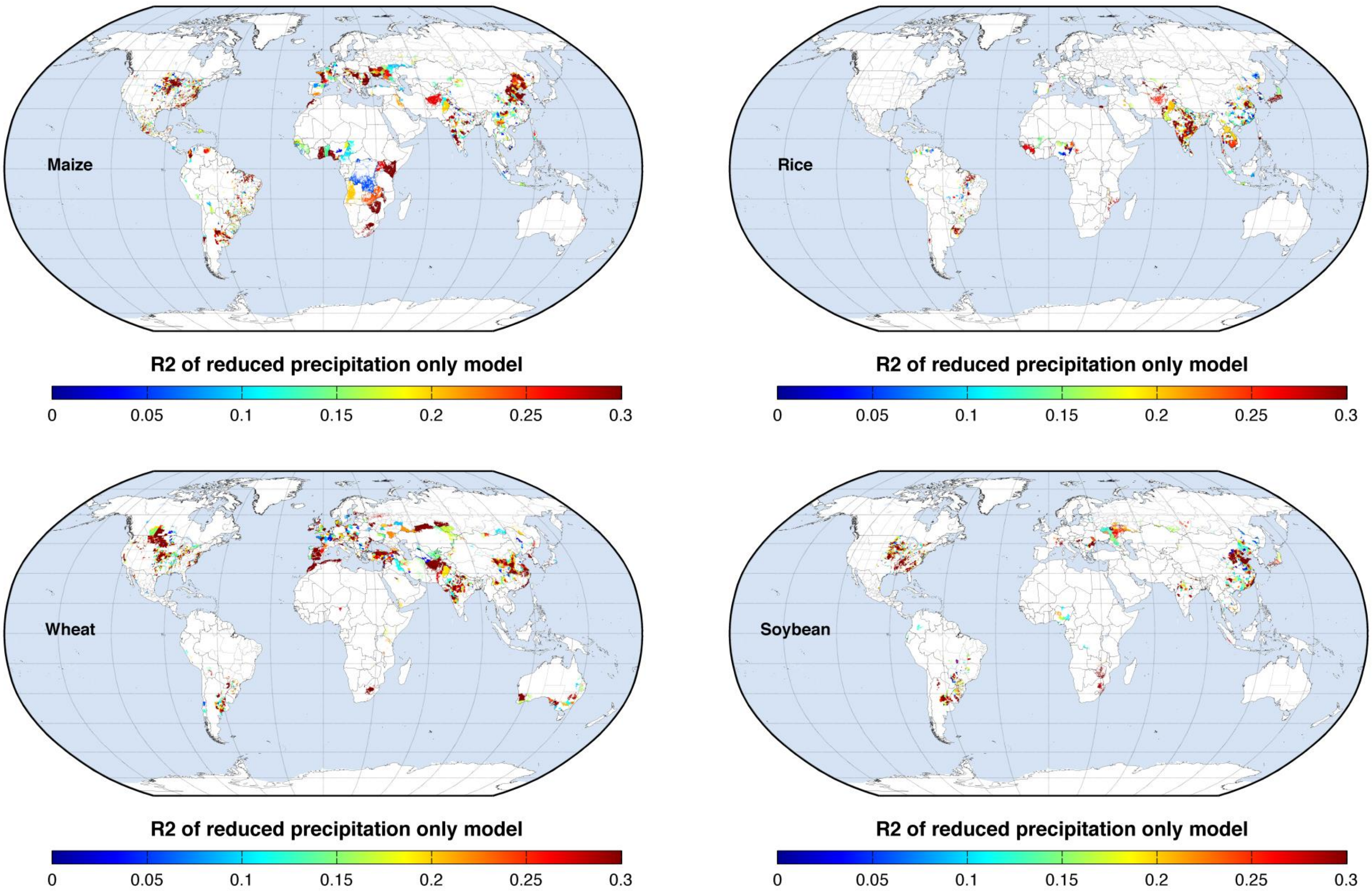

Supplementary Figure 3. **Coefficient of determination of reduced precipitation models (r_P^2).** White areas indicate where crop is not harvested or analysed.

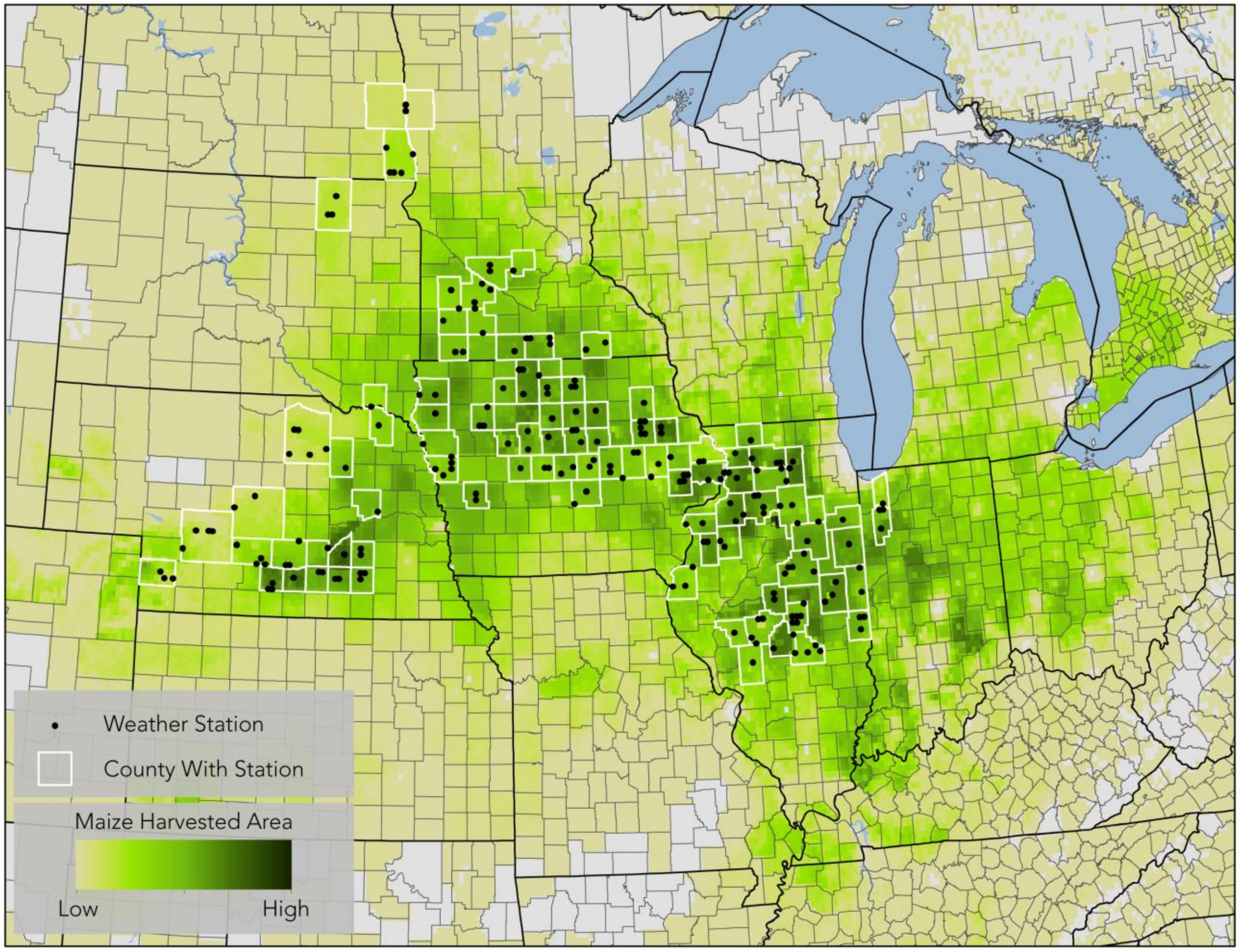

Supplementary Figure 4. **Location of weather stations with respect to where maize is harvested in the top ~100 United States maize production counties.** All weather stations used are shown even though each station did not always have continuous weather monitoring and reporting. Counties analysed are shown with white polygons. All the top maize producing counties did not have weather stations. The weather stations have a sparse covering necessitating the use of statistically corrected gridded weather data that provide complete coverage.

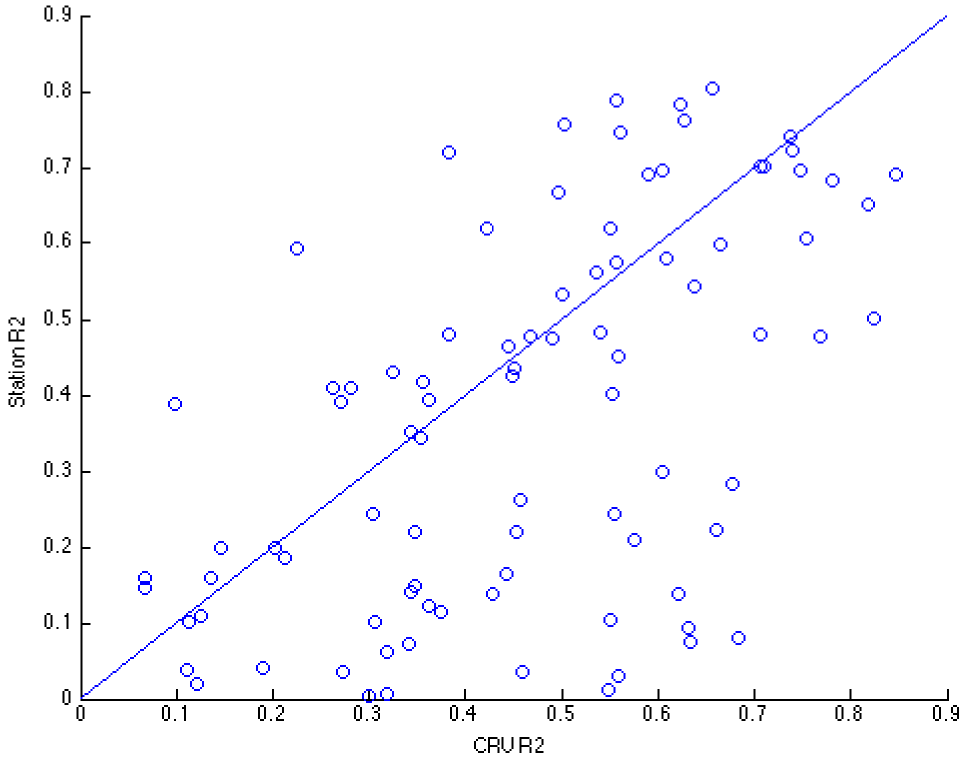

Supplementary Figure 5. **Scatter of station weather and CRU weather derived year-to-year yield variability explained by year-to-year variation in climate.** The metric being compared is the R^2 or coefficient of determination. The correlation coefficient is 0.54 at p<0.0001 (~100 counties). The CRU points are typically below the 1:1 line.

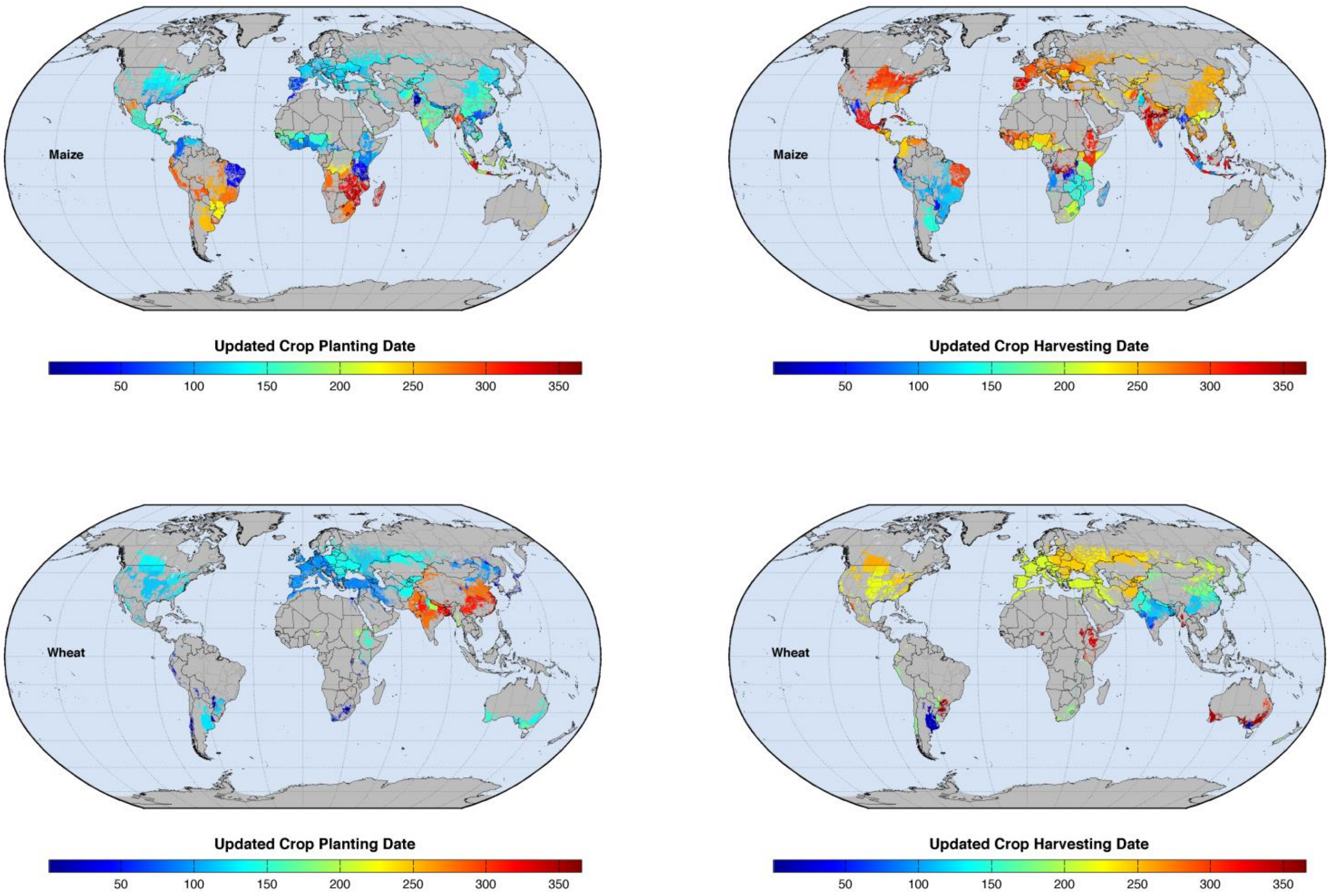

Supplementary Figure 6. **Updated main season crop planting and harvested dates for maize and wheat.** The legend shows Julian days, or January 1 is day 1, and December 31 is represented as day 365. Gray values indicate regions where crop is not harvested. Left panels show planting dates and the right panels show the harvesting dates.

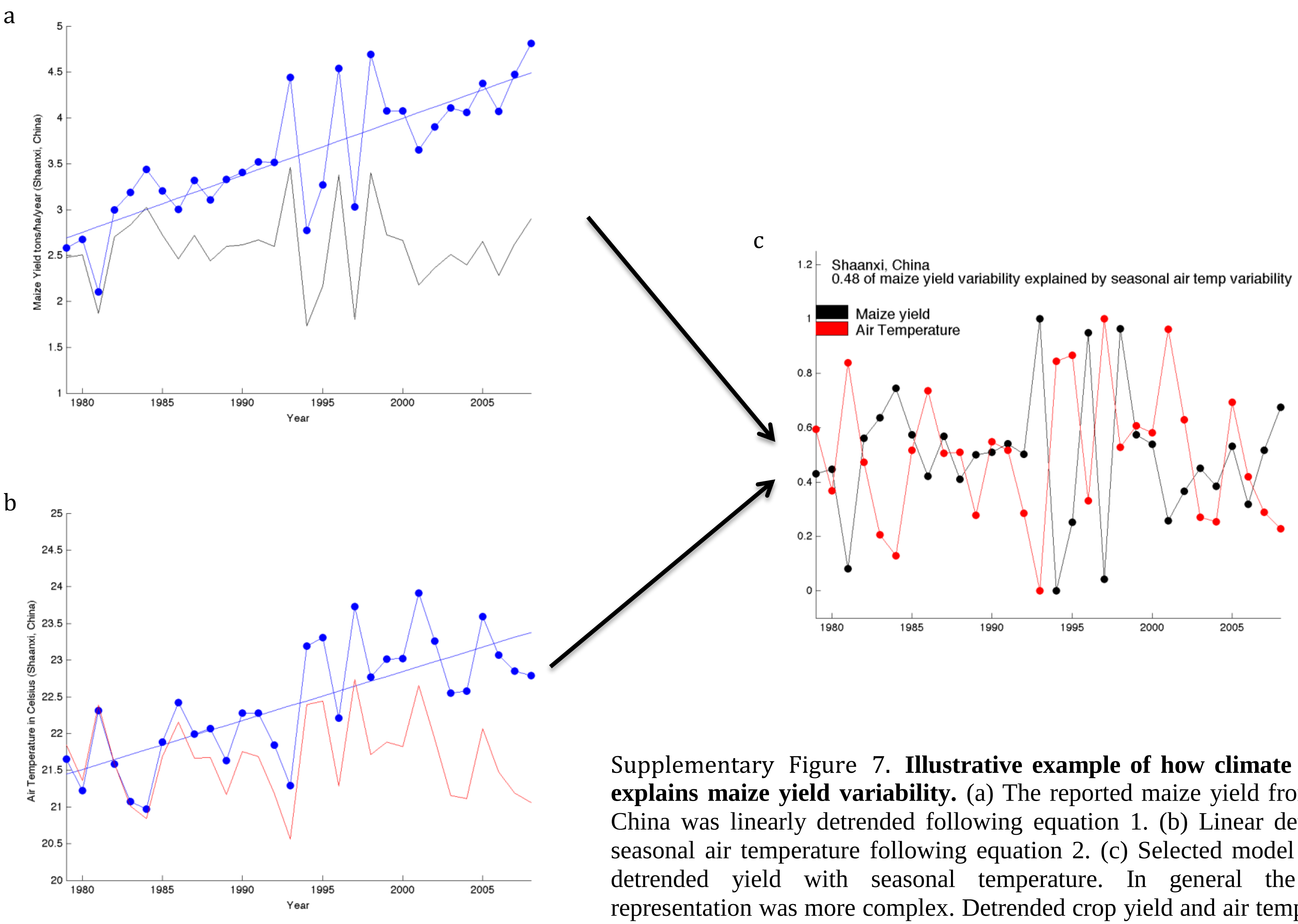

Supplementary Figure 7. **Illustrative example of how climate variability explains maize yield variability.** (a) The reported maize yield from Shaanxi, China was linearly detrended following equation 1. (b) Linear detrending of seasonal air temperature following equation 2. (c) Selected model related the detrended yield with seasonal temperature. In general the statistical representation was more complex. Detrended crop yield and air temperature are scaled between 0 and 1 – y-axis (minimum and maximum values represent 0 and 1) in the figure (c) for ease of representation; x-axis is the year.

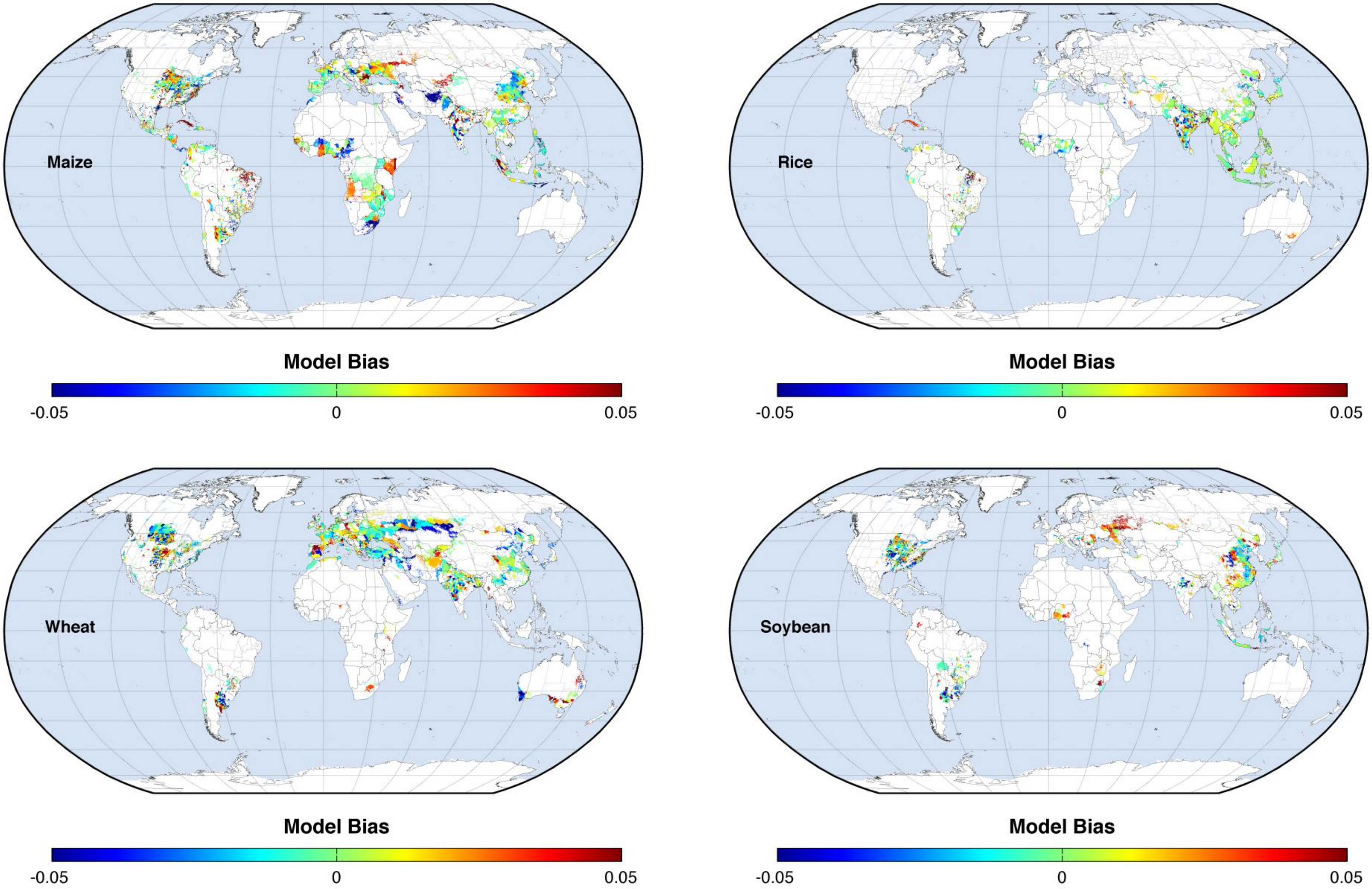

Supplementary Figure 8. **Model bias.** The bias is scaled and thus the legend reflects a bias of -5% to +5%. White areas indicate where crop is not harvested or analysed.

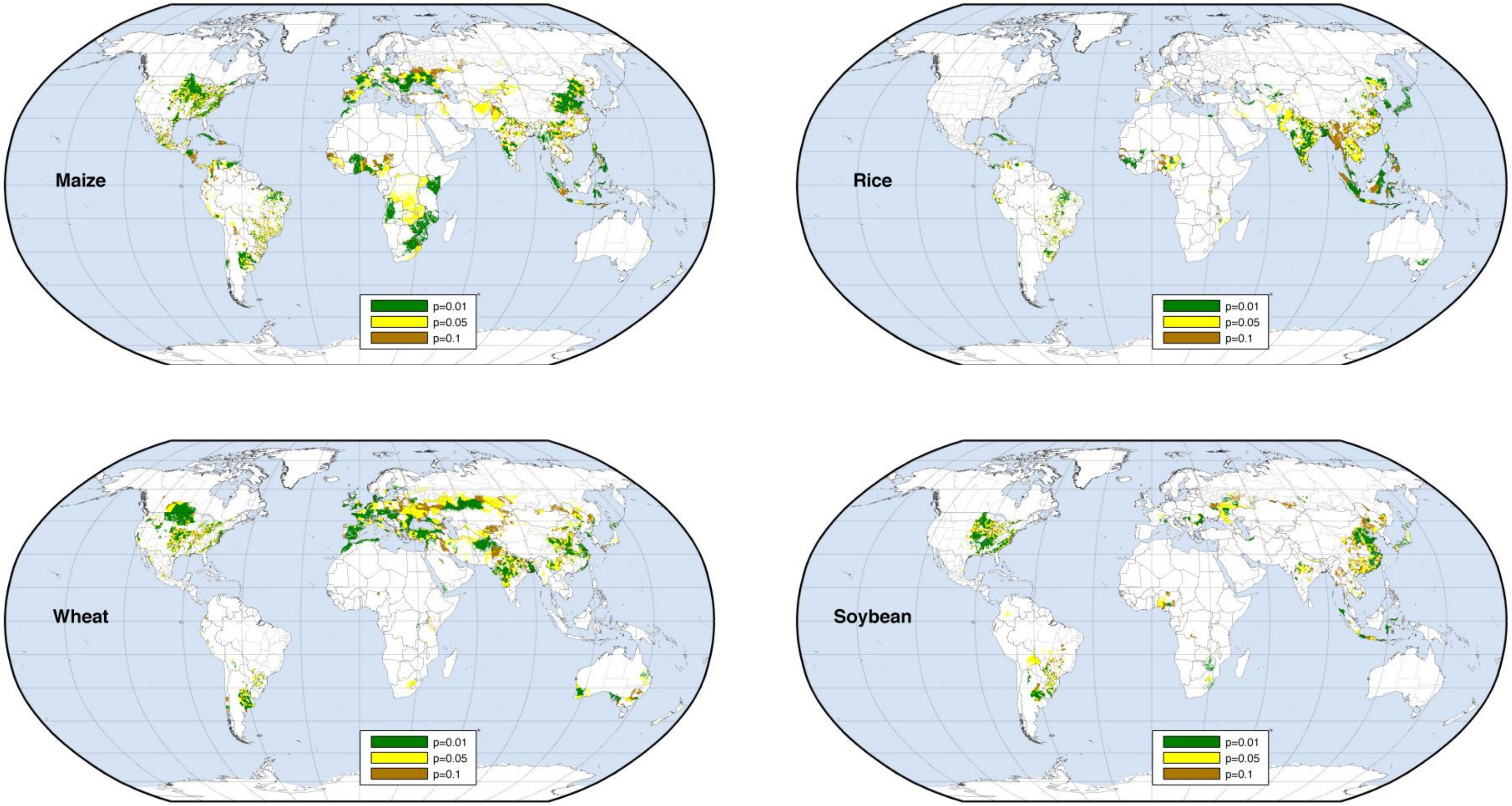

Supplementary Figure 9. **Model significance levels as determined from conducting *F*-tests against a random climate model.** Regions in green had p=0.01 (sample size study period of 3 decades per political unit; *F*-test) or models at 99% significance; yellow areas had significance between p=0.01 and p=0.05 or 99% to 95% significance (study period of 3 decades per political unit; *F*-test). Most of the regions had models at p=0.05 level. We additionally had included some more areas with p values between p=0.05 and p=0.1 or 90% to 95% confidence. White areas indicate where crop is not harvested or analysed.

Supplementary Table. TERMS PRESENT IN EACH MODEL AND RESULTING MODEL CLASSIFICATION

	temperature terms				precipitation terms				interaction terms					Model Classification
model#	St	At	St*St	At*At	Sp	Ap	Sp*Sp	Ap*Ap	St*Sp	At*Ap	St*St*Sp*Sp	At*Ap	At*At*Ap*Ap	
1	x													normal temp
2		x												normal temp
3	x	x												normal temp
4	x		x											normal + extreme temp
5		x		x										normal + extreme temp
6	x	x	x	x										normal + extreme temp
7			x											extreme temp
8				x										extreme temp
9			x	x										extreme temp
10					x									normal precip
11						x								normal precip
12					x	x								normal precip
13					x		x							normal + extreme precip
14						x		x						normal + extreme precip
15					x	x	x	x						normal + extreme precip
16							x							extreme precip
17								x						extreme precip
18							x	x						extreme precip
19	x		x		x		x		x		x			Complex
20		x		x		x		x				x	x	Complex
21	x	x			x	x			x	x				Complex
22			x	x			x	x			x		x	Complex
23	x	x	x		x	x	x		x		x	x		Complex
24	x	x		x	x	x		x	x	x			x	Complex
25	x		x	x	x		x	x	x		x		x	Complex
26		x	x	x		x	x	x		x	x		x	Complex
27	x	x	x	x	x	x	x	x	x		x	x	x	Complex

St: Seasonal temperature
At: Annual temperature
Sp: Seasonal precipitation
Ap:Annual precipitation

Supplementary Methods 1: Data

1.1 Climate data

For the climate and weather conditions influencing crop growing conditions we used the CRU TS3.10 [1] dataset. This dataset has a long history of development [2-4] and has been extensively used in numerous climate related studies including land and agricultural studies [5-9]. Version TS3.10 [1] adds more station data that CRU acquired recently. However station observations covering the world from the varied data sources still requires homogenization and interpolation of the data to a grid of 0.5 degree in latitude and longitude as explained in [1]. Other comparable climate datasets that have been used in agricultural studies are from the University of Delaware [http://climate.geog.udel.edu/] and the Global Precipitation Climatology Centre [10, 11]. TS3.10 and the UDEL temperature product, and TS3.10 and GPCC precipitation products are highly correlated. Lobell et al. [2011] [12] found similar results using either the CRU or the UDEL global climate dataset; hence we felt that using only the CRU TS3.10 was sufficient to conduct this study.

The spatial resolution of our gridded crop data (see next section) is at 5 minutes globally. Thus, within each half degree grid cell of the CRU TS3.10 data are several crop grid cells. We assigned each of the 5 minute crop grid cell that was contained within a half degree CRU grid cell the identical reported weather value. Those that lay partly within more than a single CRU grid cell were assigned the value of the nearest CRU grid cell. However note that the analysis was done at the political unit level which can contain numerous CRU grid cells. What we computed was the harvested-area-weighted temperature and precipitation information for all of the grid cells within a political unit each year; this was the political unit's climate value per month for any specific year.

1.2 Crop data

The crop yield and area harvested information came from the newly developed dataset noted in three of our previous reports [13-15]. In view of the importance of the Russian Federation especially for wheat and maize production we updated Russian maize and wheat statistics at the oblast / county level using an approach similar to those described in Ray et al. [2012] [14] specifically for this study; otherwise the rest of the crop data used is similar to the ones reported in [14, 15].

The crop statistics database provides information on crop yield and harvested areas for each of the four crops: maize, rice, wheat, and soybean at the 5 minute spatial resolution. The yield and harvested information does not distinguish between the primary crop and secondary crops such as rice or soybean grown in double cropping systems. Similarly, crops such as wheat that are grown as winter or spring wheat are not distinguished. There are several other agronomic distinctions in cropping systems, such as irrigated crops, different cultivars, upland and lowland

crops that would likely have different levels of sensitivity to climate variability. We did not make these distinctions in our crop database. The original dataset covers the period of 1961 to 2008 and contains ~2.5 million unique statistics of maize, rice, wheat, and soybean harvested and yield information. We used the period 1979 to 2008 for this study which contained ~2 million reported statistics on these four crops including the updated information for the Russian Federation [16-18].

1.3 Planting and harvesting dates

We used the main growing season determined by the average planting and harvest date for each crop as given in [19]. The crop planting and harvest dates have varied in the last three decades due to climate as well as technological and socioeconomic changes at each political unit [20]. In a perfect modeling system we would have the planting and harvesting dates as a function of time. At this point we have not built a dataset reflecting temporal crop planting and harvest dates. Moreover, the climate information is monthly, reducing sensitivity of the analysis to planting and harvesting dates. However, we did test the sensitivity of results at the top maize producing U.S. counties using daily station data and found statistically significant correlation (at $p = 0.0$) between monthly and daily climate data based analysis. In most global locations a few days advancement or delay in crop planting and harvesting still leads to the same month. Thus we used fixed crop planting and harvested dates as used in other studies [12, 21].

We however updated the global maps of crop planting and harvest dates reported in [16] based on more recent information for some of the important agricultural countries such as China [22], India [23], Pakistan [24], Bangladesh [25], Nepal [26], Australia [27], South Africa [27], Brazil [17, 28] and Argentina [17, 29] (Supplementary Figure 6). We updated the planting and harvest dates for only maize and wheat. Rice crop appeared generally more accurate globally but note that in several global locations there is a significant second rice growing season. Our utilization of weather conditions 1 year prior to harvest of the main rice crops provides information regarding rice growing conditions for the secondary season. In future studies a more precise delineation of the main and the secondary growing season yields and weather conditions needs to be modeled.

Supplementary Methods 2: Statistical analysis

2.1 Setting up of the statistical models

We set up the statistical models in three steps. We first computed the harvested-area-weighted average seasonal and average annual (12 months prior to harvest month including the harvest month) temperature and precipitation information at

each of the political units that harvested a crop (maize, rice, wheat, and soybean). In the next second step we detrended the climate and yield information by taking out the linear trends for climate and the best fit yield model following [12 and 14] for yields:

$$Y_d = Y_* + Y - Y_b \quad (1)$$
$$T_d = T_* + T - T_l \quad (2)$$
$$P_d = P_* + P - P_l \quad (3)$$

where Y, T, and P are the observed yield, temperature and precipitation, and the subscript 'd' denotes the detrended values, 'l' denotes the linear fit, and 'b' denotes the best fit from among constant, linear, quadratic, and cubic fit. The best fit in turn was determined using AIC [30]. The * denotes the initial value similar to detrending noted in [12].

We used the crop yield trend modeling described in Ray et al., 2012 [i.e. reference 14] but other investigations into crop yield modeling are also present. Specifically in [14] comparisons with the statistical method presented in another study [e.g. 30], which in turn were further investigations from other recently published methods [31,32] was shown. The yield modeling method presented in [14] was applied to national level data to compare against the national scale yield modeling presented in [30] and gave similar results. Yield modeling however continues to be an area of active research [33,34], but their performance at our level of high spatial resolution globally remains unknown, as other studies have been either regional studies or national scale studies. Future studies could investigate these developing techniques of yield modeling in global analysis of yield variability.

Only those political units that harvested a crop (maize, rice, wheat, and soybean) at least half of the years of the study period entered into our analysis. This means that a political unit needed to harvest maize crop more than 15 out of the maximum 30 years to enter into our maize analysis and so on.

In the third step we built linear regressions connecting Y_d with the single and squared forms of T_d and P_d. Note, that there are both seasonal and annual forms of the climate variables. Thus, eight types of climate variables are possible and 27 different forms of:

$$Y_d = f(T_d, P_d) \quad (4)$$

were generated. We had a sufficiently large number of permutations to cover a large set of statistical relations between the observed crop yields variability and climate variability.

Since we tracked ~13,500 political units this meant ~1.5 million statistical equations were generated for the four crops. We identified the best fit equation using the Akaike Information Criterion (AIC) [35] from the 27 equations at each

political unit which resulted in using the best ~50,000 equations to draw on our results and conclusions for the four crops. Supplementary Figure 7 gives an example of this process from Shaanxi province, China. Supplementary Figure 1 provides the average distribution of crop yields.

2.2 Determining yield variability explained by climate variability

From the 27 regression equations the equation with the lowest AIC was further analyzed. Even though the model chosen fit the data best it may still be a poor model and no better than a model that related the variability in crop yields to random climate variations or a null model. Thus we conducted an ANOVA or *F*-test at the $p = 0.10$ level to determine whether the chosen model was statistically significantly better than a random climate model. Only in those political units where the chosen model was statistically significant at the $p = 0.10$ or less level we further reported how much of the yield variability was explained by climate variability. Political units where the p value was greater than 0.10 indicated that there was no statistically significant relationship in crop yield variability and climate variability. We classified these political units as those where we could not detect statistically significant climate variability impacts on crop yields as “no effect” in Figure 2 in the main text.

The coefficient of determination, or explained variance for the chosen model, i.e. the one with the lowest AIC (equation 4), is statistically the ‘complete model’ explained variance or R^2 which is plotted as categorical maps in Figure 2 in the main text. Reduced models of temperature and precipitation were also generated with only temperature and precipitation terms. If the selected model had only temperature terms then only the reduced model for temperature was generated and vice versa. The reduced models give an indication of how important temperature and precipitation variability was in explaining yield variability and the R^2 of these models are given in Supplementary Figures 2 and 3.

2.3 Global maps

Even though the global maps are plotted at the grid cell the analysis was conducted only per political unit and the result for the political unit mapped onto all the grid cells. The bias of the selected model at each political unit, and for each crop, is plotted in Supplementary Figure 8.

One could also choose Bayesian Information Criterion (BIC) as the criterion to identify the best equation from the 27 regression equations [36], but we continued to use AIC similar to our previous studies [14,15]. Political units where crop yields completely failed would not report any harvested areas and production. These areas evidently would be undergoing an extreme event and these situations of total crop failure would not enter into the current analysis as harvested areas reported would

then be zero. The numbers reported in the summary data table for each country and crop are weighted by harvested area. Further Supplementary Figure 9 provides the spatial locations of p values of the selected models for two increasingly higher confidence limits (p=0.05 and p=0.01). The models selected at the less restricted p-value of 0.1 generally included models at p=0.05 or less.

2.4 Sensitivity to time period analyzed

Our study period spanned the thirty years from 1979 to 2008. We also conducted separate analyses to determine how sensitive the explained crop yield variability was to the length of time studied. As expected, results were slightly different. Simulations for a twenty-five year period from 1984 to 2008 resulted in climate variability explaining 40%-45% of the overall yield variability. Over a thirty-five year period (1974 to 2008) the numbers were 29%-34%. Our second set of sensitivity analysis involved conducting thirty year analyses but starting the simulations from one and two years earlier to 1979, i.e. 1978 and 1977. In both cases the results were nearly identical to numbers for analysis starting in 1979. When simulations started off from 1978, we found that a nearly similar 32%-39% of crop yield variability was explained by climate variability and for the analysis from 1977 to 2006 the numbers were 34%-39%.

2.5 Use of gridded weather versus station data

Since the yield is measured at each political unit, the weather information should appropriately represent all crop-harvested areas within the political unit and valid for each political unit similar to the procedure used in [12]. Gridded weather data such as the CRU [1] use station data, which have sparse coverage (see for example the coverage in the top maize counties of the United States - Supplementary Figure 4) to build a complete coverage through statistical analyses. Direct use of station data, which essentially represents only a short fetch around the site of the ground station (Supplementary Figure 4), should not be used to analyze the relationship between crop yields when measured at the political unit (covering a vast area) and weather. On the other hand if the yield is measured at a specific location, for example a farmer's field, then gridded weather data should not be directly used without appropriate downscaling of the data on account of relatively coarser gridded data at this scale. A better approach would then be to use a nearby sited station weather information. Our analysis being at a political unit, the unit of yield measurement, we upscaled the gridded weather data (as described in section 1.1, above) to match the yield measurement spatial unit, a method similarly used in [12]. We however conducted an alternate study to show how different our results would have been if spatial mismatch using direct station data was used. Since we did not build the CRU data, the analysis also serves as a rough check of the CRU data.

We selected the top ~100 maize producing counties in the United States (representing ~25% of total United States maize production) to perform this additional experiment. Over the study period we accessed all the archived weather station data for all the ~100 counties [37] and used the average of all, essentially point measurements, within a county boundary to generate a seasonal and annual weather for the county. The stations in general had very poor coverage of the county maize harvested areas (Supplementary Figure 4). Note that the CRU gridded data is essentially station data with statistically estimates of the weather in non-covered areas resulting in complete coverage over all land areas [1].

Next we conducted our statistical modeling directly using the station data and determined the year-to-year yield variability explained by year-to-year weather station derived climate variability (using the coefficient of determination R^2 metric). Finally we correlated the station analysis R^2 with the CRU weather derived R^2 for all counties considered. The scatter plot (Supplementary Figure 5) shows both these R^2 per county. The correlation was significant (0.54 at $p<0.001$).

Supplementary References

1. Harris I, Jones P D Osborn T J, Lister D H 2013 Updated high-resolution grids of monthly climatic observations – the CRU TS3.10 dataset *International Journal of Climatology* DOI: 10.1002/joc.3711
2. New M, Hulme M, Jones P D (1999) Representing twentieth-century space-time climate variability. Part I: development of a 1961-90 mean monthly terrestrial climatology. *Journal of Climate* **12** 829–856
3. New M, Hulme M, Jones P D (2000) Representing twentieth century space-time climate variability. II: development of 1901–1996 monthly grids of terrestrial surface climate. *Journal of Climate* **13** 2217–2238
4. Mitchell T D, Jones P D (2005) An improved method of constructing a database of monthly climate observations and associated high-resolution grids. *International Journal of Climatology* **25** 693–712
5. Lobell D B, Field C B (2007) Global scale climate-crop yield relationships and the impacts of recent warming. *Environ. Res. Lett.* **2** 014002 doi:10.1088/17489326/2/1/014002
6. Rockström J, Falkenmark M, Karlberg L, Hoff H, Rost S, Gerten D (2009) Future water availability for global food production: The potential of green water for increasing resilience to global change. *Water Resources Research* **45:7** DOI: 10.1029/2007WR006767
7. Tao F, Yokozawa M, Liu J, Zhang Z (2008) Climate-crop yield relationships at provincial scales in China and the impacts of recent climate trends. *Climate Research* **38:1**, 83-94
8. Mekonnen M M, and Hoekstra A Y (2011) The green, blue and grey water footprint of crops and derived crop products. *Hydrol. Earth Syst. Sci.* **15** 1577-1600

9. You L, Rosegrant M W, Wood S, Sun D (2009) Impact of growing season temperature on wheat productivity in China *Agricultural and Forest Meteorology* **149** 1009-1014
10. Becker A, Finger P, Meyer-Christoffer A, Rudolf B, Schamm K, Schneider U, Ziese M (2013) A description of the global landsurface precipitation data products of the Global Precipitation Climatology Centre with sample applications including centennial (trend) analysis from 1901-present. *Earth System Science Data* **5** 71–99
11. Schneider U, Becker A, Finger P, Meyer-Christoffer A, Ziese M, Rudolf B (2013) GPCC's new land surface precipitation climatology based on quality-controlled in situ data and its role in quantifying the global water cycle. *Theoretical and Applied Climatology* http://dx.doi.org/10.1007/s00704-013-0860-x.
12. Lobell D B, Schlenker W, Costa-Roberts J (2011) Climate trends and global crop production since 1980. Science **333**, 616-620.
13. Foley JA, Ramankutty N, Brauman KA, Cassidy ES, Gerber JS, et al. (2011) Solutions for a cultivated planet. Nature 478: 337-342
14. Ray DK, Ramankutty N, Mueller ND, West PC, Foley JA (2012) Recent patterns of crop yield growth and stagnation. Nature Communications 3:1293 doi:10.1038/ncomms2296
15. Ray DK, Mueller ND, West PC, Foley JA (2013) Crop yield trends are insufficient to double global food production by 2050. *PLoS ONE* 8(6): e66428 doi:10.1371/journal.pone.0066428
16. Food and Agricultural Organization - FAOSTAT (2013) Available at http://faostat.fao.org/site/567/DesktopDefault.aspx?PageID=567#ancor (Accessed May, 2013)
17. USDA (1994) Major world crop areas and climatic profiles, United States Department of Agriculture, Agricultural Handbook No. 664, revised September 1994
18. Russian Federation Federal State Statistics Service (2013) Available from http://www.gks.ru, accessed March 2013
19. Sacks W J, Deryng D, Foley J A, Ramankutty N (2010) Crop planting dates: an analysis of global patterns. Glob. Ecol. Biogeogr. **19**, 607-620
20. Kucharik, C.J. (2006) A multidecadal trend of earlier corn planting in the central USA. *Agronomy Journal*, **98**, 1544–1550.
21. Porter JR, and Semenov MA (2005) Crop responses to climatic variation. Phil. Trans. R. Soc. B **360**, 2021-2035
22. Zhang T, and Huang Y (2013) Estimating impacts of warming trends on wheat and maize in China from 1980 to 2008 based on county level data. *International Journal of Climatology* **33** 699-708
23. Indian Council of Agricultural Research (2013) Crop Science Division. Available from http://eands.dacnet.nic.in/
24. Pakistan Meteorological Department (2013). Available from http://namc.pmd.gov.pk
25. Bangladesh Yearbook of agricultural statistics (2011), Bangladesh Bureau of Statistics 23rd edition. Available from www.bbs.gov.bd

26. Joshi N P, Maharjan K L, Luni P (2011) Effect of climate variables on yield on major food-crops in Nepal – A time-series analysis. *Journal of Contemporary India Studies: Space and Society, Hiroshima University* **1** 19-26
27. USDA (2013), major world crops areas and climate profiles. Available from http://www.usda.gov
28. Bradesco (2013) Available from http://www.bradescorural.com.br
29. Bert F E, Satorre E H, Toranzo F R, Podestá G P (2006) Climatic information and decision-making in maize crop production systems of the Argentinean Pampas. *Agricultural Systems* **88** 180-204
30. Lin M, Huybers P (2012) Reckoning wheat yield trends. Environmental Research Letters 7: 024016, doi: 10.1088/1748-9326/7/2/024016
31. Brisson N, Gate P, Gouache D, Charmet G, Oury F-X, et al. (2010) Why are wheat yields stagnating in Europe? A comprehensive data analysis for France. Field Crops Research 119: 201–212. doi: 10.1016/j.fcr.2010.07.012
32. Finger R (2010) Evidence of slowing yield growth–the example of Swiss cereal yields. Food Policy 35: 175–182. doi: 10.1016/j.foodpol.2009.11.004
33. Michel M, Makowski D (2013) Comparison of statistical models for analyzing wheat yield time series PLOS ONE 8(10): e78615. doi:10.1371/journal.pone.0078615
34. Grassini P, Eskridge KM, Cassman KG (2013) Distinguishing between yield advances and yield plateaus in historical crop production trends. Nature Communications 4: 2918
35. Akaike H (1974) A new look at the statistical model identification. *IEEE Trans. Automat. Contr.* **19** 716–723
36. Schwarz G E (1978) Estimating the dimension of a model. Annals of Statist., **6**, 461-4
37. National Climate Data Center (NCDC) available from http://www.ncdc.noaa.gov/cdo-web/
